# Faunal activity rhythms influencing early community succession of an implanted whale carcass offshore Sagami Bay, Japan

**DOI:** 10.1038/s41598-018-29431-5

**Published:** 2018-07-24

**Authors:** J. Aguzzi, E. Fanelli, T. Ciuffardi, A. Schirone, F. C. De Leo, C. Doya, M. Kawato, M. Miyazaki, Y. Furushima, C. Costa, Y. Fujiwara

**Affiliations:** 1 Instituto de Ciencias del Mar (ICM) of the Consejo Superior de Investigaciones Científicas (CSIC), Paseo Marítimo de la Barceloneta, 37-49, 08003 Barcelona, Spain; 2 Department of Life and Environmental Sciences, Polytechnic University of Marche, Via Brecce Bianche, 60121 Ancona, Italy; 3 Marine Environment Research Centre of the Italian National Agency for New Technologies and Sustainable Development (ENEA), P.O. Box 224, 19100 Pozzuolo di Lerici (SP), Italy; 4 Ocean Networks Canada, University of Victoria, PO Box 1700 STN CSC, Victoria, BC V8W 2Y2 Canada; 5 Department of Biology, University of Victoria, PO Box 3080, Victoria, BC V8W 2Y2 Canada; 6 Japan Agency for Marine-Earth Science and Technology (JAMSTEC), 2-15 Natsushima-Cho, Yokosuka, Kanagawa 237-0061 Japan; 7 Consiglio per la ricerca in agricoltura e l’analisi dell’economia agraria (CREA), Centro di ricerca per l’Ingegneria e le Trasformazioni agroalimentari (CREA-IT), 00016 Monterotondo, Italy

## Abstract

Benthic community succession patterns at whale falls have been previously established by means of punctual submersible and ROV observations. The contribution of faunal activity rhythms in response to internal tides and photoperiod cues to that community succession dynamism has never been evaluated. Here, we present results from a high-frequency monitoring experiment of an implanted sperm whale carcass in the continental slope (500 m depth) offshore Sagami Bay, Japan. The benthic community succession was monitored at a high frequency in a prolonged fashion (i.e. 2-h intervals for 2.5 months) with a seafloor lander equipped with a time-lapse video camera and an acoustic Doppler profiler to concomitantly study current flow dynamics. We reported here for the first time, to the best of our knowledge, the occurrence of strong 24-h day-night driven behavioral rhythms of the most abundant species (*Simenchelys parasitica*; *Macrocheira kaempferi*, and *Pterothrissus gissu*). Those rhythms were detected in detriment of tidally-controlled ones. Evidence of a diel temporal niche portioning between scavengers and predators avoiding co-occurrence at the carcass, is also provided. The high-frequency photographic and oceanographic data acquisition also helped to precisely discriminate the transition timing between the successional stages previously described for whale falls’ attendant communities.

## Introduction

The fate of large whale carcasses sinking to the seafloor in both shallow and deep-sea environments has intrigued scientists in several research fields spanning from community ecology, evolution, and biogeography for three decades^1–4^. The term ‘whale fall’, coined after the incidental discovery and observations of a balaenopterid whale skeleton at a depth of 1240 m in Santa Catalina basin (California, USA), has been since used to generally define these oases-like organic-enriched seafloor environments^1,2^. In extremely food-limited environments such as the deep sea, a 40-ton sunken adult whale provides an enormous input of organic matter (~2 × 10^6^ g C), that may be equivalent to over 200 years of particulate organic matter arriving at the seafloor *via* vertical and lateral fluxes in the form of marine snow aggregates and phytodetritus^2^.

Whale carcasses not only provide a surplus in food supply to benthic and demersal animals belonging to a vast array of trophic guilds, but they also serve as habitats for a specialized fauna that represents the counterpart of chemosynthetic symbiont-bearing taxa, inhabiting other reducing environments such as hydrothermal vents and hydrocarbon seeps^1,2,5^. Anaerobic bacterial decomposition of lipid-rich content within whalebone matrices releases sulfide into the environment, which is taken up by sulfur-oxidizing chemoautotrophic bacteria found as endosymbionts of several invertebrate species free-living in the seafloor^3^. Because of that resemblance and the sharing of several species with hydrothermal vents and cold seeps, whale falls have been hypothesized to act as stepping-stone dispersal pathways between those habitats^1–3,5^. At evolutionary time scales, whale carcasses have also been postulated to support the invasion of deep-sea chemosynthetic habitats by shallow water taxa, also in a stepping-stone fashion^5–7^.

After three decades of studies on naturally occurring and artificially implanted whale carcasses on the seafloor, four stages of ecological succession were established (see 2 and 3 for details): *i*. the mobile-scavenger stage, lasting months up to 1.5 years, *ii*. an enrichment-opportunistic stage, lasting months up to 4.5 years, *iii*. a sulphophilic stage, lasting for decades, and finally *iv*. a reef stage from remaining bones, potentially lasting also for several years. The duration of each stage is ultimately dependent upon the local rate of whale carcass decomposition, which defines the lipid content of specific bones along the skeleton^8^. Additionally, seafloor environmental conditions such as depth, current speeds, temperature, and dissolved oxygen have also been postulated to influence in the duration of whale fall community successional stages^2,9^.

Cyclic changes in the environmental conditions around sunken whale carrion may deeply influence the benthic community succession dynamic, by generating a rhythmic turnover in local species composition. In the aphotic deep sea, changes in water currents and temperature^10,11^, combined with the seasonal nature of food supply from the euphotic zone^12–14^, are the main environmental drivers of temporal behavioral rhythms of benthos. Three main types of rhythmic population displacements have been well characterized: *i*. up and down the water column occurring typically at diel (i.e. 24-h) intervals (diel vertical migration); *ii*. horizontal displacements along the seabed (nektobenthic movements across shelves and slopes); *iii*. in and out from the sediment-water interface (endobenthic)^15^. The role of those population displacement rhythms on whale carcass scavenging dynamics has been poorly investigated to date. The behavioral activity of scavenging species and their access to the whale carrion could be temporally regulated by internal tidal motions, as well as upon the rhythmic presence of benthopelagic predators in the local benthic boundary layer (BBL)^15^. These rhythmic displacements all combined could in turn affect the dynamics of energy dispersal from the center of the carcass towards the background seafloor in the form of particulate and dissolved organic matter, and also fecal material (as predicted and modeled by^16^. Interspecific competition between facultative *versus* obligated scavengers could also play a crucial role in regulating species turnover near sunken whale carcasses. These two trophic groups may have developed a temporal niche partitioning strategy, accessing the carrion at different times during a 24-h diurnal period^17,18^, to avoid competition.

Previous studies on whale fall communities have relied on punctual sampling and observations conducted by submersibles and Remote Operated Vehicles (ROVs)^1,2,4,6,9,19^. High-frequency *in-situ* observations and sampling are currently inexistent due to logistical constraints, mostly associated with high operational costs in accessing remote deep-sea environments. Furthermore, the obvious difficulties associated in locating naturally occurring whale falls, as well as the laborious logistics involved in artificially implanting whale carcasses in the deep seafloor contribute to the current lack of detailed observations^20^. Nevertheless, multiparametric seabed monitoring platforms such as landers are promising tools that have never been used for the purpose of studying sunken whale carcasses in the deep-sea. Specifically, landers equipped with video cameras and a suit of oceanographic sensors can be efficiently used to study the contribution of species activity rhythms to the scavenging dynamics, and to better constrain the transition time scales between previously defined community successional stages.

The present contribution furthers our knowledge on whale fall community succession at much finer temporal scales, particularly at the initial mobile-scavenger stage. It also provides new insights on how scavenging activity dynamics can also be regulated by species-specific behavioral rhythms that in the aphotic Deep-sea should be chiefly modulated by diurnal/semi-diurnal internal tides. We employed a chronobiology approach in data collection and time-series analyses (see^21^, for a methodological review). All benthic scavenging megafauna occurring at and near an artificially implanted whale carcass at the upper slope (492 m) off Sagami Bay, Japan were counted at a bi-hourly frequency over a 2.5-months. Periodogram, waveform, and multivariate statistical analyses were employed to evidence how patterns of diel and weekly fluctuations in species’ visual counts (as proxies of population displacement rhythms) are related to the oceanographic conditioning. We also used seafloor temperature and current flow data from the lander to perform a Fast Fourier Transform (FFT) analysis, identifying relationships between individual species counts and overall environmental variability.

## Materials and Methods

### The study site and the experimental setting

**Figure 1:**
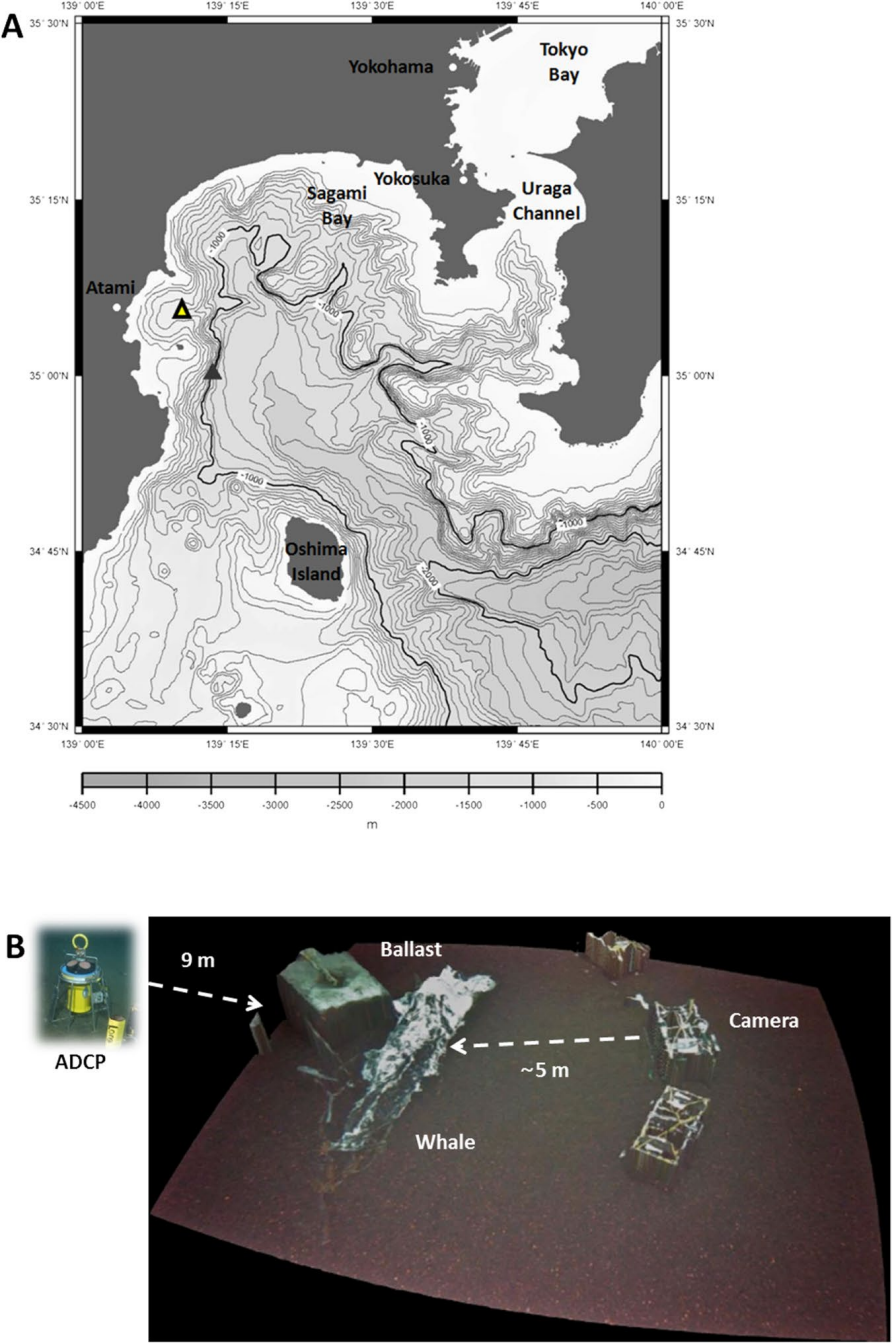
Map showing the deployment site of the juvenile sperm whale SW off Sagami Bay (large triangle) at 500 m depth and in relation to the JAMSTEC seafloor cabled video-observatory (small black triangle) at 1100 m depth (source 22). Elaborated with QGIS 2.18 (https://www.qgis.org/it/site/).

A juvenile male sperm whale (*Physeter macrocephalus*, Linnaeus 1758), 464 cm in total length and ca. 1.2 tons of body weight, was collected whole as it beach-stranded at Mihama-chō, Aichi, Japan on April 24^th^, 2008. The carcass was preserved in a freezer container at −30 °C until June 5^th^, 2012. The whale was then transported to the Atami Port and stored on a barge that was used for the carcass deployment. The free-fall deployment of the carcass was then conducted at a depth of 492 m at the halfway point between Hatsushima Island and the tip of the Manazuru Peninsula in Sagami Bay (35°05.576′N, 139°10.271′E) on June 8^th^, 2012 (Fig. 1). For selecting the deployment location, three practical issues had to be considered. The deployment depth must be shallower than 1000 m because the seabed lander was transported by the HOV *Triton 3300* that had the maximum operational depth at 1000 m. At depths shallower than 1000 m fishery is allowed, therefore, we had to negotiate with local fisheries’ unions and finally had received the permission for the deployment from the unions around Atami City. Additionally, there are large inaccessible areas in the bay, due to the occurrence of submarine cables. Further, the selection of the deployment site took in consideration the slope disphotic depth character, to allow both the study of day-night and tidal rhythms, as well as the contribution of large shallow water predators (e.g. sharks) on the early scavenging dynamic^9^.

Two concrete blocks (1 m^3^ each) for ballast were tied to the base of the whale tail by negatively-buoyant polypropylene ropes (45 mm in diameter). The carcass riggings followed safety recommendations from the Japanese Fisheries Agency (http://www.jfa.maff.go.jp/j/whale/w_faq/pdf/manual.pdf), and the deployment was documented in video by the Japan Broadcasting Corporation (NHK) and by the Discovery Channel (http://www.godac.jamstec.go.jp/catalog/data/doc_catalog/media/NT12-15_all.pdf).

The seabed lander used to carry out imagery acquisition was equipped with a time-lapse still imaging system (Goto Aquatics Inc., Japan), consisting of a digital camera Pen Lite E-PL2 (Olympus, Japan), a Zuiko Digital ED 8 mm F3.5 Fisheye lens (Olympus), and two incandescent strobe flashlights FL-50R (Olympus). These were installed on both sides of the camera, coupled with a diffuser (GN28, ISO200). The fisheye lens and curved dome port of the camera housing allowed for a very wide and large field of view. The exposure of the camera was manually set to 1/125 sec for the shutter speed and to f/16.0 for the aperture. The white balance was set to 5500 K and the focal distance was set to 120 cm.

The camera was installed facing forward (i.e., in the same horizontal plane of the seafloor), with the height of the center of the lens at 42 cm above the seafloor, and distanced approximately 5 m away from the carcass. With those camera settings, the entire field of view was estimated in 10 m^2^, encompassing the entire whale carcass and the seabed in the foreground. Photographs were acquired at 2-h intervals for a period of 72 days, starting on June 14^th^ at 16:30 (6 days after carcass implantation) and ending on August 24^th^ at 10:30 (Japan Standard Time, JST), when the camera system was retrieved using the ROV *Hyper-dolphin*. During the video-monitoring period, sunrise and sunset shifted their timing from 4:24 to 5:07, and from 18:57 to 18:19, respectively (https://www.timeanddate.com/sun/japan/tokyo?month=6&year=2012). Image acquisition started 6 days following the carcass because of the bad weather and mechanical problems to HOV’s manipulator.

Image acquisition was supplemented by the monitoring of oceanographic conditions such as bottom currents and temperature around the carcass. Besides providing key information of environmental fluctuation potentially influencing faunal presence as proxy of activity rhythms, the characterization of seafloor currents is also useful to study the intensity and direction of odor plumes released by the decomposing carcass^2,16^,^22^. Additionally, temperature variability has also been assigned as a key regulator of activity rhythms in ectotherms^23–25^. A 300 kHz Workhorse Sentinel Acoustic Doppler Current Profiler (ADCP; Teledyne RD Instruments) was installed upward-looking in the southwestern side of the whale carcass, providing current velocity (horizontal and vertical components), temperature, and hydrostatic pressure (water column height, as proxy of depth) data. The instrument was programmed to record data at a 20-minute sampling interval, with sample bin size and number of bins set to 3 and 50 m, respectively. We focused our observations on the first layer above the transducer (5 m from the bottom), to target the hydrodynamic variations in the BBL environment. Current flow velocities were analysed for the North-South (N-S), East-West (E-W), and vertical components, being used to describe the tidal influence in the overall local current regime, and after employing a Fast Fourier Transform (FFT) analysis. Bottom current and temperature data have been down sampled into 2-h intervals to match the frequency of the still imagery acquisition.

### Time-series data analysis of the physical environment and benthic fauna

We estimated the fluctuations in benthic megafauna visual counts derived from the still imagery to be used as a proxy of local population rhythm, resulting from synchronic displacement among individuals^26,27^. For each image, we identified and counted animals to the lowest taxonomic level as possible, reporting abundance numbers per each operational taxonomic unit. We could only identify animals that were approximately within 4–5 m^2^ of the field of view in front of the camera, with a few exceptions represented by large-size species.

The occurrence of significant diel periodicities in visual count fluctuations for the different species (i.e., modulated by either day-night or internal tide cycles) was studied using the Lomb-Scargle Periodogram included in the El Temps chronobiology software package (www.el-temps.com). A period is defined as the temporal lag between consecutive peaks and troughs in a time series. All periodicities in oceanographic and species count time series were screened within 600–1620 min intervals, equivalent to 10 and 27 h, respectively, covering a wide range of diel cycles (i.e. inertial, tidal, and of photoperiod, all associated with the latitude and depth of the study area; see^22^. In the periodogram output plot, the highest peak exceeding the significant threshold (p < 0.05), represents the maximum percentage of total data variance explained by the inherent dominant periodicity^21^.

The phase of a rhythm represents the timing of an average-peak in relation to an external controlling cycle^21^. After periodogram computing, the waveform analysis was employed to identify all rhythms’ phases only for those species showing significant diel periodicities in the prior periodogram analysis. According to^28^, each time series was partitioned into 24-h segments (i.e. 12 values per segment, given the 2-h sampling frequency). Values at corresponding timings were then averaged within all segments, thus obtaining a mean fluctuation plot (i.e. the waveform). Phase timing and duration were statistically assessed using the Midline Estimating Statistic Of Rhythm (MESOR)^29^. MESOR was estimated by re-averaging all waveform values and representing the resulting mean as a horizontal threshold line on the waveform plots. This threshold was used to discern waveform values above it as a significant increment representative of the phase^21^.

For those species showing significant visual count patterns with tidal periodicity, waveform analysis was repeated as above, but at an approximated 12-h length (i.e. by subdividing the whole data set in sub-segments, each of 6 values), to evidence the role of bottom current flow on species behavior patterns. Additionally, to detect the occurrence of tidal motions and to analyze their temporal evolution, a Fast Fourier Transform (FFT) analysis was performed on the time-series of horizontal and vertical velocities. Thus, the waveform and FFT analyses combined, were also repeated in a similar manner for the ADCP data using the first sample bin (i.e. 5 m above seabed), indicating tidal motions. A comparison between the two approaches was also performed. The 24-h Fourier component of E-W current was also integrated in time to evaluate the periodic displacements along this direction induced by diel tidal current. Thus, the E-W component was selected as representative of the horizontal flow, being oriented approximately towards the main direction of the bottom topography.

The overall time-series analysis (i.e. periodogram combined with waveform) was repeated by subdividing species according to their main feeding habits: scavengers (i.e., carrion eaters, which can also be facultative predators), detritivores (i.e., animals that feed on dead decomposing organic material, including plant-based detritus), and predators (i.e., eating live prey). Visual counts for species within each trophic group were summed at 2-h intervals, and time series analyses were repeated, and results compared.

### Multivariate statistical analyses

Multivariate analyses were carried out to define the timing of community succession, as revealed by temporal changes in single species occurrences. First, a cluster analysis with a similarity profile (SIMPROF) test^30^ was carried out on the week-averaged 4^th^-root transformed biological data matrix, in order to highlight separation in species occurrences within each observation period. Second, a non-metrical Multi-Dimensional Scaling (nMDS) analysis was performed on the same matrix, and bubble plot charts used to highlight the species mostly contributing to the observed ordination. A Permutational Multivariate Analysis of Variance (PERMANOVA)^31^ was carried out on the taxonomic groups found to be significantly different in terms of visual counts by the SIMPROF test. Finally, a Similarity Percentages (SIMPER) analysis was run to identify those species that most typify each week and the average similarity within each period (week). All these analyses were carried out with the software PRIMER6 & PERMANOVA+ add-on packages^31,32^.

In order to determine the relative contributions of each oceanographic parameter on temporal patterns of individual species counts, and in the occurrence of certain recurrent species associations, a Canonical Correspondence Analysis (CCA) and a 2-Block Partial Least Squares analysis (2B-PLS) were carried out^33^. A matrix composed by the time series observations (from 18:30 of June 14^th^ to 4:30 of August 22^th^ 2012 for a total of 822 observations) and 2 series of variables (i.e. “block” hereafter) were considered. Block 1 was composed by 5 variables: Date (numerically transformed in Excel), Time (also numerically transformed in Excel and shifted starting from 4:30 a.m. as “dawn”, to obtain lower values in the photo phase and high values at night), and finally temperature, depth, and current velocity (horizontal E-W and vertical current components). Again, the E-W component was selected as representative of the horizontal flow, as approximately follows the main direction of the bottom topography (see Fig. 1). Block 2 was composed by variables as species visual counts and block 3 encompassed trophic-group variables of scavengers (and facultative predators), detritivores, and predators. Being an ordination technique, CCA was then applied to observe in a same multidimensional space the contributions of both environmental and species count matrices. The ordination axes are linear combinations of the environmental variables. CCA and 2B-PLS analyses were performed using the free software PAST 2.17c (http://folk.uio.no/ohammer/past/).

No experiments have been conducted on live vertebrates. All methods were carried out in accordance with relevant guidelines and regulations and all experimental protocols were approved by Japan Agency for Marine-Earth Science and Technology (JAMSTEC).

## Results

### Benthic and scavenger community composition

**Figure 2:**
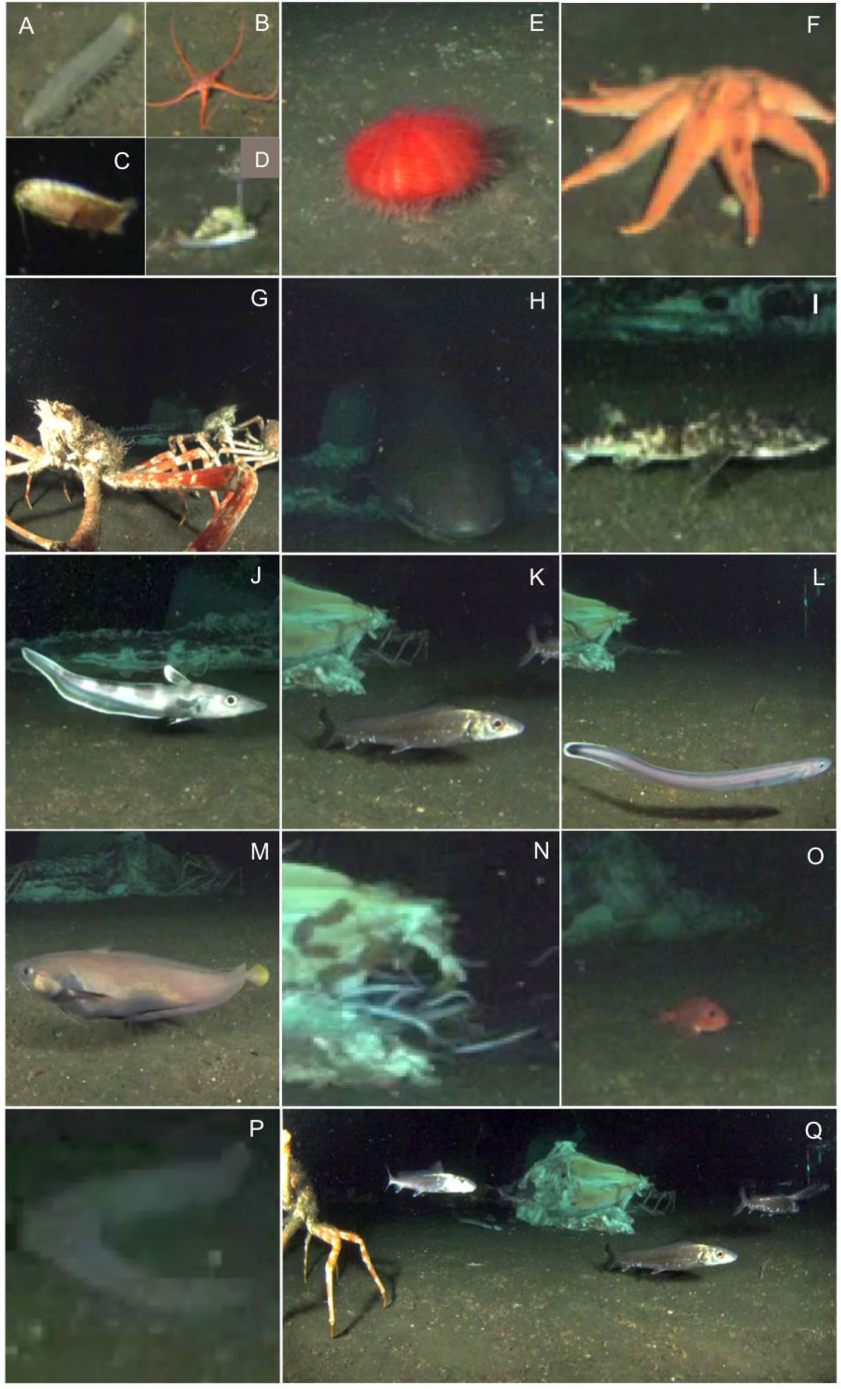
Images at different scales of the most abundant megafauna species (identified to the lowest taxonomical rank as possible), occurring at or near the whale carcass (see also Table 1): (**A**) *Pannychia moseleyi*; (**B**) Ophiurida; (**C**) *Bathynomus doederleini*; (**D**) *Buccinum yoroianum* (**E**) Echinothurioida; (**F**) *Solaster paxillatus*; (**G**) *Macrocheira kaempferi*; (**H**) *Hexanchus griseus*; (**I**) *Cephaloscyllium umbratile*; (**J**) *Coelorinchus* sp.; (**K**) *Pterothrissus gissu*; (**L**) Zoarcidae not identified; (**M**) *Physiculus japonicus*; (**N**) *Simenchelys parasitica* (various individuals feeding at the carcass); (**O**) *Helicolenus hilgendorfii*; (**P**) *Eptatretus deani*; (**Q**) broad view of the carcass with several individuals of *P. gissu* and one individual of *M. kaempferi* leaving the field of view - left of the frame. The poor quality of some of the images indicates the loss in resolution after extracting frame grabs from the video.

A total of 850 photographs were used for counting and identifying organisms. Vertebrates and invertebrates from four different phyla (Mollusca, Arthropoda, Echinodermata, and Chordata) were observed with markedly different abundances (Table 1). Sixteen putative species could be distinguished and counted (Fig. 2), while no records for macro-infaunal organisms were reported given our field of view and camera resolution settings.

**Table 1:** Taxonomical assemblage detected over 72 days starting on the 14^th^ June 2012 are hierarchically listed by phylogeny and grouped according to behavioral traits (type of movement and life habit as relationship with the seabed), along with bibliographic sources used to derive such an information.

Taxonomical Unit	Movement	Life Habit	Trophic Habit	References	N	%	P		Var.
**Echinodermata**									
*Pannychia moseleyi*	Walker	Epibenthic	Detritivore	^47, 48^	62	0.86	—	—	—
Echinothurioida	Walker	Epibenthic	Detritivore	^49^	21	0.29	—	—	—
*Solaster paxillatus*	Walker	Epibenthic	Scavenger (and predator)	^50^	73	1.02	—	—	—
Ophiurida	Walker	Endobenthic	Detritivore	^51^	755	10.52	—	—	—
**Mollusca**									
*Buccinum yoroianum*	Walker	Epibenthic	Scavenger (and predator)	^22, 52^	9	0.13	—	—	—
**Arthropoda**									
*Bathynomus doederleinii*	Swimmer	Endobenthic	Scavenger	^53^	349	4.86	—	—	—
*Macrocheira kaempferi*	Walker	Epibenthic	Scavenger (and predator)	^43, 54^	1002	13.96	1430	*23.8*	7.76
**Chordata**									
*Hexanchus griseus*	Swimmer	Nektobenthic	Scavenger (and predator)	^40, 55^	2	0.03	—	—	—
*Cephaloscyllium umbratile*	Swimmer	Epibenthic	Predator	^56^	4	0.06	—	—	—
*Caelorinchus* sp.	Swimmer	Epibenthic	Predator	^57^	16	0.22	1170	*19.5*	8.56
*Pterothrissus gissu*	Swimmer	Benthopelagic	Predator	^58, 59^	390	5.43	1440	*24.0*	22.64
Zoarcidae	Swimmer	Epibenthic	Predator	^22^	189	2.63	—	—	—
*Physiculus japonicus*	Swimmer	Benthopelagic	Predator	^60^	220	3.07	740	*12.3*	7.78
*Simenchelys parasitica*	Swimmer	Nektobenthic	Scavenger	^61^	4052	56.46	1440 (1410)	*24.0*	15.79
*Helicolenus hilgendorfi*	Swimmer	Epibenthic	Predator	^6^	9	0.13	—	—	—
*Eptatretus deani*	Swimmer	Epibenthic	Scavenger (and predator)	^35^ ^,62^	24	0.33	—	—	—
Assemblage (Total)					7177	100	1410 (1440)	*23.5*	11.06
Scavengers (and predators)					5511	76.80	1440 (1410)	*24.0*	12.92
Detritivores					838	11.70	—	—	—
Predators					828	11.50	1440	*24.0*	22.07

The number of individuals per species (N), their relative abundances (%), periodogram analysis outputs as significant (*p* < 0.05) periodicities (P) in minutes (min) and hours (h) are reported along with periodogram peak variance (Var., %) as proxy of rhythm strength. In the periodicity column, we also reported in parenthesis significant sub-periodicities as a proxy of weak tidal patterning. Total animal abundance by functional groups with respect to the 3 trophic groups is also provided.

The snubnosed parasitic eel, Simenchelys parasitica, was the most abundant species, representing more than half of total observations (56.5%, 4052 individuals) (see Table 1). The Japanese spider crab, Macrocheira kaempferi, was the second most abundant species, present in 14.0% of the observations and with 1002 individuals, followed by another bony fish, Pterothrissus gissu with 5.4% in frequency of occurrence and 390 individuals (see Table 1). Finally, the isopod Bathynomus doederleinii was the fourth most abundant species with 4.9% (349 individuals) of occurrence.

The detection at the beginning of the photograph time-lapse sequence of a large individual of the bluntnose six-gill shark, Hexanchus griseus, feeding on the whale carcass was noteworthy (Fig. 2H). The whole whale carcass slightly shifted its position at the beginning of the survey (i.e. 6:30 on June 15^th^), with 3 additional position shifts during the experiment, at 8:30 on July 15^th^, 4:30 on July 26^th^, and finally at 2:39 of the on August 7^th^. In all these occasions carcass movements could have been produced by the scavenging activity of large shark individuals, although there was no evidence from the time-lapse photographs.

The visual count time series for all species indicate different patterns of occurrence near the carcass (Appendix 1), depicting a noticeable change in community composition particularly marked by the slow replacement of the crab *M. kaempferi* by the fish *P. gissu* around the 34^th^ day of the experiment (between the 4^th^ and the 5^th^ week). While some species occurred during the entire period of observations (e.g. *S. parasitica*), others peaked toward the middle (e.g. the isopod *B. doederleini*) or the end (e.g. ophiuroids and the sea cucumber Pannychia moseley) of observations.

### Time series analysis output

For the whole testing period, average (±sd), maximum, and minimum values for the measured oceanographic variables were: temperature (°C) = 5.73 ± 0.44, max. = 7.00, min. = 4.23; pressure (dBar) = 493 ± 0.49, max. = 493.68, min. = 491.31; vertical velocity component (cm/s) = −1.89 ± 1.2, max. = 3.7, min. = −5.6; north-south velocity component (cm/s) = −4.9 ± 5.8, max. = 14.9, min. = −42.4; east-west velocity component (cm/s) = 1.47 ± 13.5, max. = 51.2, min. = −30.3.

**Figure 3:**
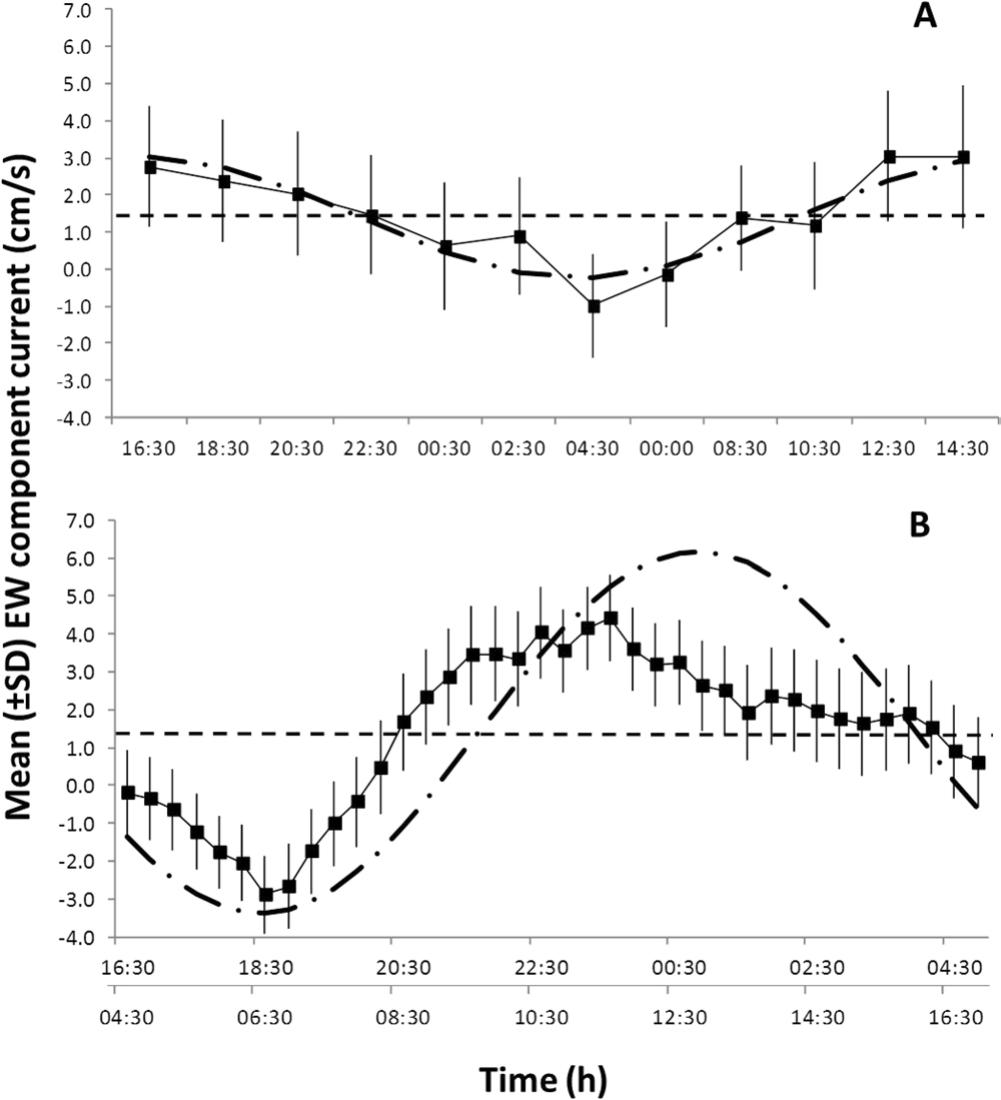
Waveform analysis output for E-W current component data. (**A**) Time-series partitioned in 24-h segments highlighting both diurnal and semidiurnal tides. (**B**) Time-series partitioned in 12-h segments (resulting in a two-row X-axis). Note that the sampling frequency for the bottom current data (20 min) have been down-sampled to match the frequency of the still imagery acquisition, or 2-h (see Methods). The MESOR is represented by the dashed horizontal line. The semidiurnal and diurnal Fourier component curves are superimposed (the tick dash-dotted lines), showing general accordance with the waveform analysis.

The analysis of the vertical component of current velocity data showed the presence of a prominent downward flow in each vertical layer, especially stronger near the bottom, and following a semidiurnal period directly linked with the tides (Appendix 2A). While peak downward currents occurred during spring tides, the weakest currents coincided with neap tide conditions. A mixed diurnal-semidiurnal tidal cycle occurred in the area, as indicated by the FFT analysis (Appendix 2B). In all the time series the semidiurnal (12-h) cycle was dominant, with lower cycles present at 6 and 24-h periods. Using the FFT diel (i.e. 24-h) component of the E-W currents, it was possible to evaluate the water displacement associated to the tidal motions by integrating the E-W component over time. This allowed us to estimate both the distance travelled by this flow (ranging between 0 to 440 m), and its time phase when it reached the maximum distance (i.e. 2-h, Fig. 3A). This overall current flow displacement provides important information about the potential maximum distance over which an odor plume originating from the whale carcass would travel (not considering diffusion), being therefore able to attract scavengers. The correspondent integrated displacements along the E-W axis in each semi-period were 440, 940, and 140 m, corresponding to 24, 12, and 6-h, respectively. These lengths may be considered as the average distances reached by each cyclic movement of tidal (diurnal and semidiurnal) periodicity for the flow around the seafloor. Similar results have been obtained for water temperature data (but with a less evident peak in correspondence of the 24-h period).

The periodogram analysis identified the occurrence of significant day-night related (i.e. 24-h) visual count fluctuation patterns for 3 species (see Table 1): *S. parasitica* (swimmer; P = 24.0 h; Var. = 15.79%), *P. gissu* (swimmer; P = 24.0 h; Var. = 22.64%), and finally *M. kaempferi* (walker; P = 23.8 h; Var. = 7.76%). A single species showed a tidal-related periodicity, the fish *Physiculus japonicus* (swimmer; P = 12.3 h; Var. = 7.78%). Interestingly, no other species showed significant tidal periodicity and only *Coelorinchus* sp. showed a different inertial currents related patterning (P = 19.5 h.; Var. = 8.56%).

The comparisons between the waveform and FFT analysis for current data are shown in Fig. 3. The waveform analysis has only been shown for the E-W current components, partitioned in 12 and then in 24-h segments to show semidiurnal and diurnal tides, as both tidal components were in phase with the vertical current velocity component. Hence, both components produced oscillations along the local seafloor topography. The correspondent diurnal and semidiurnal Fourier component curves (dash-dotted lines superimposed in Fig. 3) confirmed the same trends. Both the vertical and E-W current velocity components showed an evident bimodal fluctuation, typical of mixed diurnal and semidiurnal regime, even if the semidiurnal cycle was clearly dominant (about tenfold the diurnal cycle).

**Figure 4:**
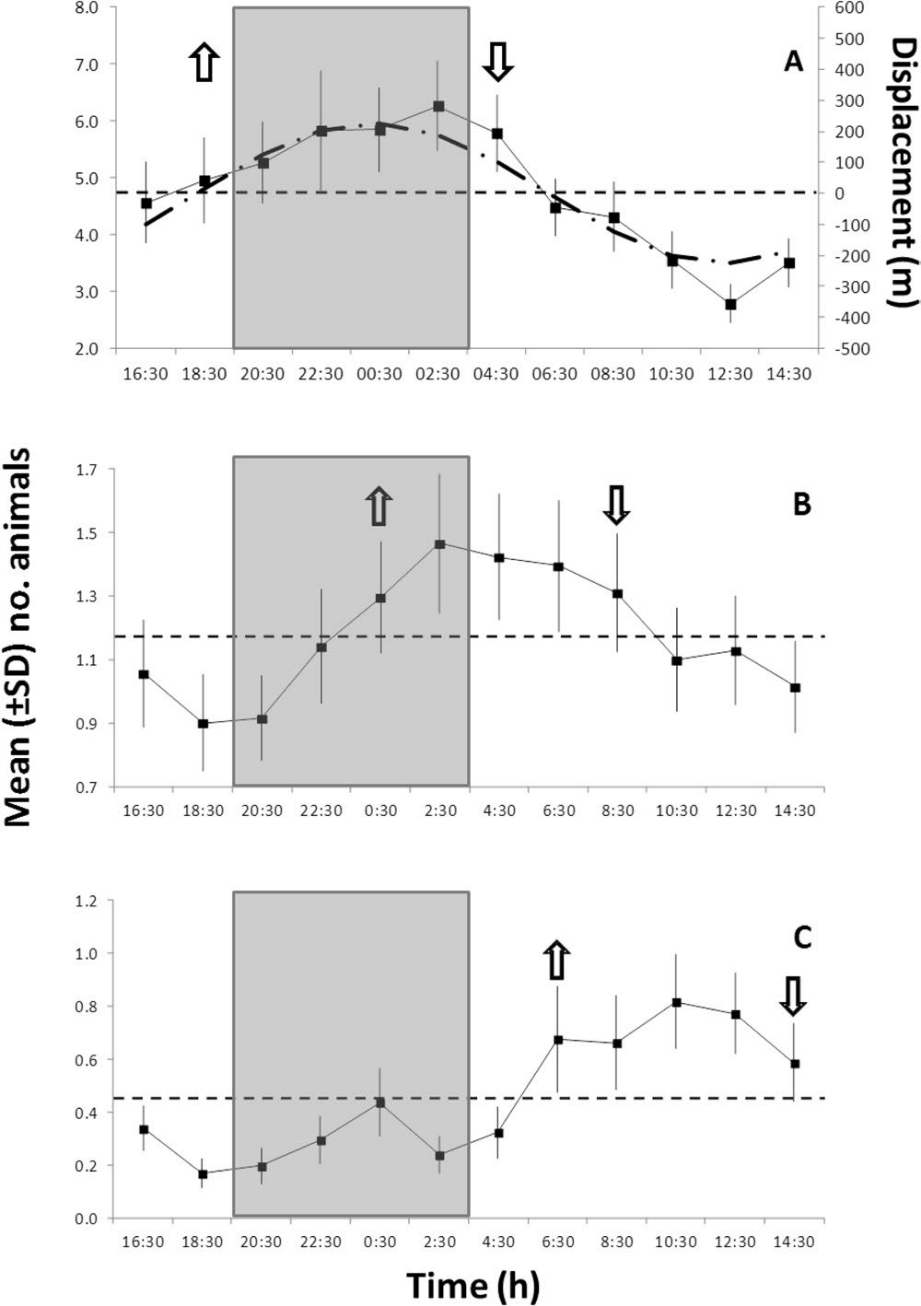
Waveform analysis output for the visual count time series of species showing significant 24-h periodicities in the periodogram analysis (see Table 1). (**A**) *Simenchelys parasitica*; (**B**) *Macrocheira kaempferi*; and (**C**) *Pterothrissus gissu*. Note the different Y-axis scales, reflecting variable visual count ranges among species. Up and down arrows indicate onset and offset of significant increments in abundance peaks, respectively. They also indicate the first and last calculated mean abundance values above the Midline Estimated Statistic Of Rhythm (MESOR; thin dashed horizontal line). MESORs are: (**A**) 4.76; (**B**) 1.18; and (**C**) 0.46. The correspondent integrated diurnal current flow displacements (in meters) along the E-W axis, computed from the FFT analysis, are superimposed (tick dash-dotted line) to *S. parasitica*, showing a 2**-**h delay in the animal periodicity. Grey rectangle depicts approximated night duration at the latitude of the study site.

The waveform analysis conducted for *S. parasitica*, *P. gissu*, and *M. kaempferi* showed significant 24-h periodicity based on the visual count patterns of the previous periodogram analysis (Fig. 4, Table 1). This indicated the occurrence of temporally coherent phases among those three species (i.e. a continuous series of values above the MESOR; see Methods) and a progressive phase shift from night (*S. parasitica*) to daytime (*P. gissu*). The integrated values of diurnal flow displacements along the E-W axis, computed from the FFT results, are superimposed: *S. parasitica* phase shows a delay of 2 hours, while for the *M. kaempferi* such a delay is of about 4 hours.

**Figure 5:**
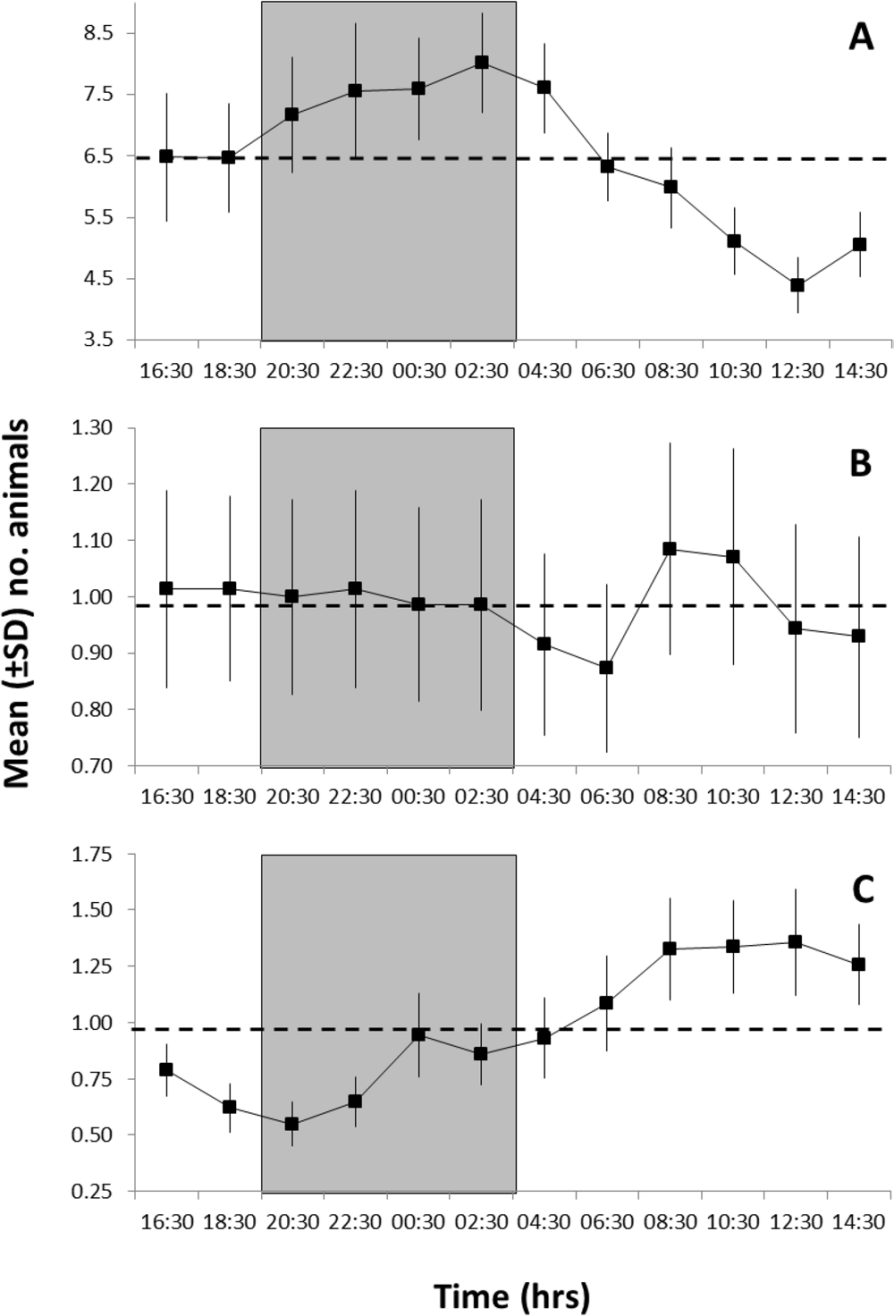
Waveform analysis output for the visual count time series of: (**A**) scavengers (and facultative predators); (**B**) detritivores; and finally, (**C**) predators. Plots indicate the occurrence of significant abundance peaks shifted in time (i.e. values above the MESOR as horizontal dashed line). MESORs are: (**A**) 6.48; (**B**) 0.99; (**C**) 097. Grey rectangle depicts approximated night duration at the latitude of the study site.

The waveform analysis conducted separately for scavengers, detritivores, and predators, indicated the presence of a temporal segregation of species near the carcass according to their main feeding group (Fig. 5). While scavenger and facultative predator abundance was phased at night (Fig. 5A), predators were more abundant during the day (Fig. 5C). Differently, detritivores did not show a clear trend in abundance throughout the day, with only a phase with minor amplitude was present in the morning, likely due to highly variable visual counts from species belonging to this trophic group. These results were corroborated by the periodogram analysis output (see Table 1), which identified significant 24-h rhythmicity for scavengers and predators as well as arrhythmia for detritivores.

### Multivariate statistical analyses

Two main phases of benthic community succession at the whale fall, were identified by the cluster analysis at 60% of similarity level: weeks 1–6 significantly separated from weeks 7–11 (SIMPROF test, *p* < 0.05; black lines in Appendix 3A). The clustering of weeks 1–6 and 7–11 was corroborated by the PERMANOVA test (*F*_*1,10*_ = 11.34, *p* < 0.001).

The SIMPER analysis further identified those species and higher taxa that mostly typified each of the two phases of the early whale fall community succession (Table 2). Overall, while *S. parasitica* was abundant throughout the entire observation period (i.e., weeks 1–11), *M. kaempferi* characterized the whale all community during weeks 1–6, and unidentified ophiuroids during weeks 7–11. These results were further supported by the nMDS bubble plots (Appendix 3B).

**Table 2:** Results of the SIMPER analysis carried out on two periods (weeks 1–6 *vs*. 7–11) based on the Bray-Curtis dissimilarity matrices.

Species	Av. Abund.	Av. Sim.	Contrib. (%)	Cum. (%)
**Weeks 1–6**	**Average similarity: 72.08**			
*S. parasitica*	1.37	36.87	51.16	51.16
*M. kaempferi*	0.86	19.11	26.51	77.67
*P. japonicus*	0.25	5.37	7.45	85.11
*B. doederleini*	0.27	3.91	5.43	90.55
**Weeks 7–11**	**Average similarity: 75.77**			
Ophiurida	1.03	26.33	34.75	34.75
*S. parasitica*	0.92	23.74	31.33	66.08
*P. japonicus*	0.23	6.16	8.12	74.2
Zoarcidae	0.25	5.95	7.85	82.06
*M. kaempferi*	0.28	5.87	7.74	89.8
*P. gissu*	0.3	5.58	7.37	97.17

**Figure 6:**
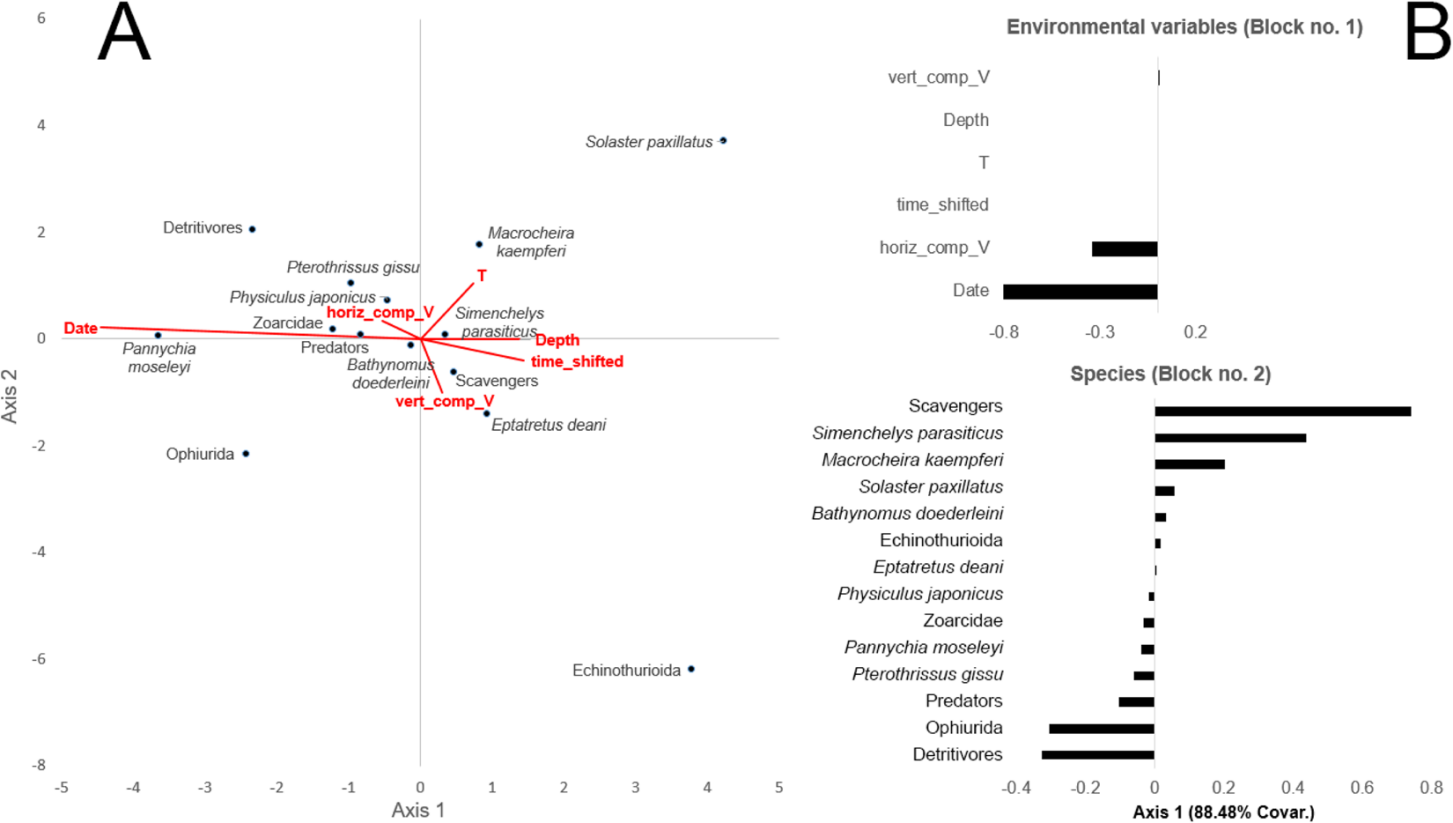
Multivariate statistic results. (**A**) Scatter plot of the first two axes of the CCA analysis. Species and trophic categories (i.e. scavengers, predators, and detritivores) are represented by black points and environmental variables are represented by red arrows (i.e., vectors). The environmental variables considered were date of the observation, spatial components of current velocity (“EW_Comp_V”, “Vert_Comp_V”), depth, temperature, and the time (numerically transformed and shifted so to start from 4:30 as “dawn”). (**B**) Bar chart of the score loadings relative to the first latent vector (explained variance = 77.44%) for both environmental and species blocks (no. 1 and 2, respectively).

The CCA analysis showed the association between species (represented by points, block 2) with the environmental variables (represented as vectors, block 1) (Fig. 6A). The first ordination axis (horizontal) segregates species by timing of appearance (variable ‘Date’) at the whale carcass (i.e., early successional species appear towards the right of the axis, and later stage species towards the left). These results are in agreement with the periodogram, waveform, cluster, and nMDS analyses. Additionally, they highlight the late appearance of detritivores such as ophiuroids and holothurians at the carcass, marking a transition between successional stages. From right to left in that ordination axis we have scavengers, predators, and finally detritivores. The 2-block PLS (Fig. 6B) showed how the most important variables on the first axis of the block 1 (88.5% Covariance) were primarily the date, followed by the horizontal component of the current velocity.

## Discussion

We reported here for the first time, to the best of our knowledge, the occurrence of a diel turnover in the species assemblages scavenging and foraging at and in the vicinities of an artificially implanted whale carcass at mid slope depths off Japan. This result alone provides some new insights into the role of benthic and demersal species’ behavioral rhythms in affecting whale fall community succession and energy dispersion into deep-sea slope environments. Additionally, we also provide evidence for a diel temporal niche partitioning among species with clearly different foraging strategies, which avoid co-occurrence (i.e. feeding at the carcass) during the same time periods of the day. The high-frequency photographic and oceanographic data acquisition, using an autonomous lander, also helped to precisely discriminate the transition timing between previously described community successional stages at whale falls; i.e., from the initial ‘mobile-scavenger’ stage, to the second ‘enrichment-opportunist’ stage (*sensu* 2).

Surprisingly, strong day-night related behavioral rhythms of the most abundant species occurring at the whale carcass (*Simenchelys parasitica*; *Macrocheira kaempferi*, and *Pterothrissus gissu*) were detected in detriment of tidally-controlled rhythms. In a region where a diurnal- mixed with a dominant semi-diurnal internal tide current regime is well characterized (Appendix 3B), we expected that animals undergo large synchronized benthopelagic displacements following those high-energy tidal currents^22^. Furthermore, at those depths in the slope (i.e., approx. 500 m), we expected that animal behavior would be largely decoupled from photoperiod-related cues, even though the ocean disphotic layer is variable and can reach 800–1000 m^17^,^34^,^35^. Unfortunately, the lack of information on light spectrum penetration and attenuation for those slope depths in the study area off Japan prevent us from further exploring the role of photoperiodicity in controlling benthic and benthopelagic species behavioral rhythms.

The observed short-term (24-h) periodic shifts in the whale carcass community composition due to species’ activity rhythms should then be analyzed in the context of previously described massive synchronous faunal displacements along bathymetric gradients that transition between photic and disphotic depths^36^. Therefore, individual animals or schools swarming horizontally along the BBL (nektobenthic mode^11^, or vertically through the water column (benthopelagic mode^11^, may be periodically present at local deep slope BBL following a rhythmicity that is decoupled from the tidal flow drift, but rather primarily triggered by photoperiod cues experienced at shallower (photic) strata^11,22^. Furthermore, the role of photoperiod cues in determining temporal dynamics of benthic assemblage structure composition is corroborated by previous studies from day-night trawling surveys in other slope areas of central Japan, which demonstrated that benthopelagic and nektobenthic displacements are triggered by day-night alternation^37^. This important indirect effect of day-night cycles on slope benthic community composition adds another layer of temporal complexity in the structuring of deep-sea ecosystems, and here in particular of the community observed at an experimentally deployed whale carcass^11^.

Higher abundances *M. kaempferi* occurred during minimum E-W flow (see Fig. 3B), and 8 hours after the maximum correspondent current displacement (~440 m), possibly indicating maximum range in odor plume displacement. Day-night rhythmic population displacements of *M. kaempferi* near the whale carcass were somehow associated with currents, with potential cascading effects on the species’ scavenging rates and resulting energy dispersal from the carcass (in the form of particulate organic matter and fecal material, as described in^16^; albeit not directly quantified during our study).

While some benthic and demersal species or faunal groups may perform long migrations across a large depth range at continental slopes, some species display rhythmic displacements that are not yet fully characterized. That is the case for example of the highly mobile predator fish *Pterothrissus gissu* and the scavenger *Simenchelys parasitica*. Despite the fact that we identified significant diel rhythmic peaking in abundance near the implanted carcass, we still lack information if these species undergo similar nektobenthic rhythms as single individuals or sizable aggregations. The only species that showed a semidiurnal tidal-driven behavioral rhythm identified by the photograph’s visual counts was the Japanese codling *Physiculus japonicus* (see Table 1). This species, a bathydemersal fish with a wide depth distribution range from shelf to upper bathyal depths (200–1200 m^38^), seems to undergo horizontal movements back and forth along the seabed utilizing the strong E-W current flow associated with the semi-diurnal tides. Considering the estimated maximum current flow displacements from the FFT analysis, the odor plumes emanating from the whale carcass could have travelled offshore at least 940 m during each tidal cycle, reaching deeper areas of the slope and potentially attracting *P. japonicus* to the carcass. The snubnosed eel *Simenchelys parasitica*, typically living at depths from 500 to 1800 m, was the most abundant species recorded during our time-lapse photography study. The species’ peak occurrences near the carcass followed a predictable rhythmicity occurring two hours later with respect to the diel water displacement along the slope (Fig. 3); a time lag that also could indicate odor plume dispersal reaching *S. parasitica* individuals at greater depths on the slope. All the examples provided above were corroborated by the high explanatory power of the E-W horizontal current velocity component in the ordination analysis (see Fig. 6).

The progressive substitution of the most abundant species over the time span of the experiment mirrored a faunal assemblage replacement from primarily scavengers to predators, and finally by detritivore species (see Appendix 1). Close to ~50-days after the carcass implantation, a marked transition from a ‘mobile-scavenger’ to an ‘enrichment-opportunistic’ successional stage could be clearly identified^2^. This transition occurred right after most soft-tissues were removed from the whale carcass, and when a large number of ophiuroids, a successional stage indicator group^2^, started to colonize the sediments nearby the remaining whale skeleton. Important to note here is the relatively small body mass (1.2 ton) of the juvenile sperm whale carcass used in this experiment. Smith and Baco^2^ described the duration of the ‘mobile-scavenger’ stage to range between 4–5 mo to 1.5–2 yr following carcass arrival in the seafloor, depending primarily on carcass mass (using 5 and 35-ton gray whale carcasses as end-members). Therefore, it may not be surprising that for a 1.2-ton carcass, this initial shift between ecological successional stages took place much earlier, at ~1.8 months. Furthermore, our experiment took place at a shallower depth (~500 m) if compared with deployments of the majority of previously studied whale fall communities, either found accidentally or artificially implanted (~1000–4000 m)^1,2,4,19^, with the exception of a couple much shallower whale fall experiments, in 30 and 125 m depth in Norway^39^. Depth is expected to play a crucial role on overall benthic community succession near whale falls, as background community composition and relative abundances change substantially as you move from food-limited bathyal (1000–3000 m) and abyssal (3000–6000 m) settings, into food-enriched shallower shelf and slope depths (0–1000 m^1–3,19^). At these shallower depths, higher abundances of both exclusive and facultative scavengers occur, resulting in much faster arrival times and carcass consumption rates^2,3^. Consequently, a fast carcass consumption was reported within 1.8 month of observations in our study, off Sagami Bay.

Marked transitions in community succession near the carcass were evidenced throughout the ~11 weeks of time-lapse photography, which were corroborated by the CCA (see Fig. 6) and nMDS (see Appendix 3A) ordination analyses, revealing the importance of elapsed time (i.e., ‘date’ factor) as the first and most important variable structuring the whale fall community during the study. During weeks 1–4, the mobile scavengers *S. parasitica* and *M. kaempferi* dominated the whale fall community. At week 5, we observed the beginning of replacement of *M. kaempferi* by the predator *P. gissu*, while *S. parasitica* remained the most abundant species. On week 6, detritivore ophiuroids and the scavenger isopod *B. doederleinii* became more abundant, with ophiuroids substantially increasing in abundance in week 7 and until the last period of observations at week 11. Deposit-feeding/detritivore elasipodid holothurian *P. moseleyi* appeared for the first time near the carcass at week 7.5, and remained present until week 11. Zoarcid fishes and *P. japonica*, both predators, had low and somewhat constant abundances throughout the entire 11-weeks period. These observations corroborate the predictions^2,3,19,39^: a community transition from predominantly large and mobile scavengers, to smaller, less mobile, opportunist and detritivores, taking advantage of the smaller particulate organic matter generated and dispersed near the carcass by the feeding activity of large scavengers. However, now we provide, at a much finer temporal resolution (relative to punctual ROV/submersible observation studies), the exact transition times between successional stages, which could have further implications for modeling energy dispersal from large localized organic islands in the deep ocean.

One interesting but perhaps expected observation (i.e. a single photograph at the very early stage of our experiment), was the presence of the snubnose six-gill shark *Hexanchus griseus* (Day 1), devouring a large chunk of the carcass flesh (see Fig. 2H). With a wide bathymetric range in occurrence (0–2000 m^40^), *H. griseus* has been detected as scavenging on whale remains at early stages of decomposition of both natural whale falls and artificially implanted carcasses (reviewed in^2,3^). At our study site and depth, which is well within the range of *H. griseus’* bathymetric distribution, we suspect that a large individual played a role in dislodging and moving the carcass during at least one occasion (from a total of three carcass shift movements). On August 8, nearly 60 days after the experiment deployment, as the carcass remains had already lost a large proportion of its flesh consumed by scavengers, a much lighter carcass could have been easily dislodged by the feeding frenzy activity of one or multiple *H. griseus* specimens. Even though this could not be confirmed by time-lapse photographs, the absence of high bottom current velocities immediately prior to the carcass position shift, corroborate our hypothesis that large shark(s) may have played a role in dislodging the carcass. These types of observations, and the enhancement of our understanding of whale fall community succession, at much finer temporal resolution, could be further improved if we had an even higher temporal resolution in the photographic sampling rate. Something that could easily be achieved with the use of a cabled-observatory infrastructure^20,26,27^.

Another remarkable finding of our study was the evidence for a diel temporal niche partitioning among species belonging to distinct trophic groups. In essence, scavengers (*S. parasitica* and *M. kaempferi*) and predators (*P. gissu*) alternated their occurrence (and peak densities) at the carcass, with the former group peaking during the night and the latter during the day (see Fig. 5). This short-term temporal niche partitioning strategy, found in wide range of terrestrial and marine communities competing for common resources (e.g. food or habitat), results in an enhancing survivorship for the avoidance of physical conflict^18,41,42^. Here, the whale carrion was the primary food resource being partitioned, being at the same time a prey attractor during the continuous ecological successional stages^2^. Therefore, the carrion acted as two directly correlated niche dimensions: food and primal habitat. The first attracting scavengers through odor plume dispersal^43–45^, and the second attracting predators to an optimal foraging habitat^2,3^. In macrourid fishes for example, indications of intra- and interspecific temporal niche partitioning were obtained near baited cameras in abyssal settings (reviewed in^45^). Fish individuals tended to leave unconsumed bait to avoid competition when foraging opportunity dropped below the average level for the background environment, supporting the framework of optimal foraging theories^43,45,46^.

**Figure 7:**
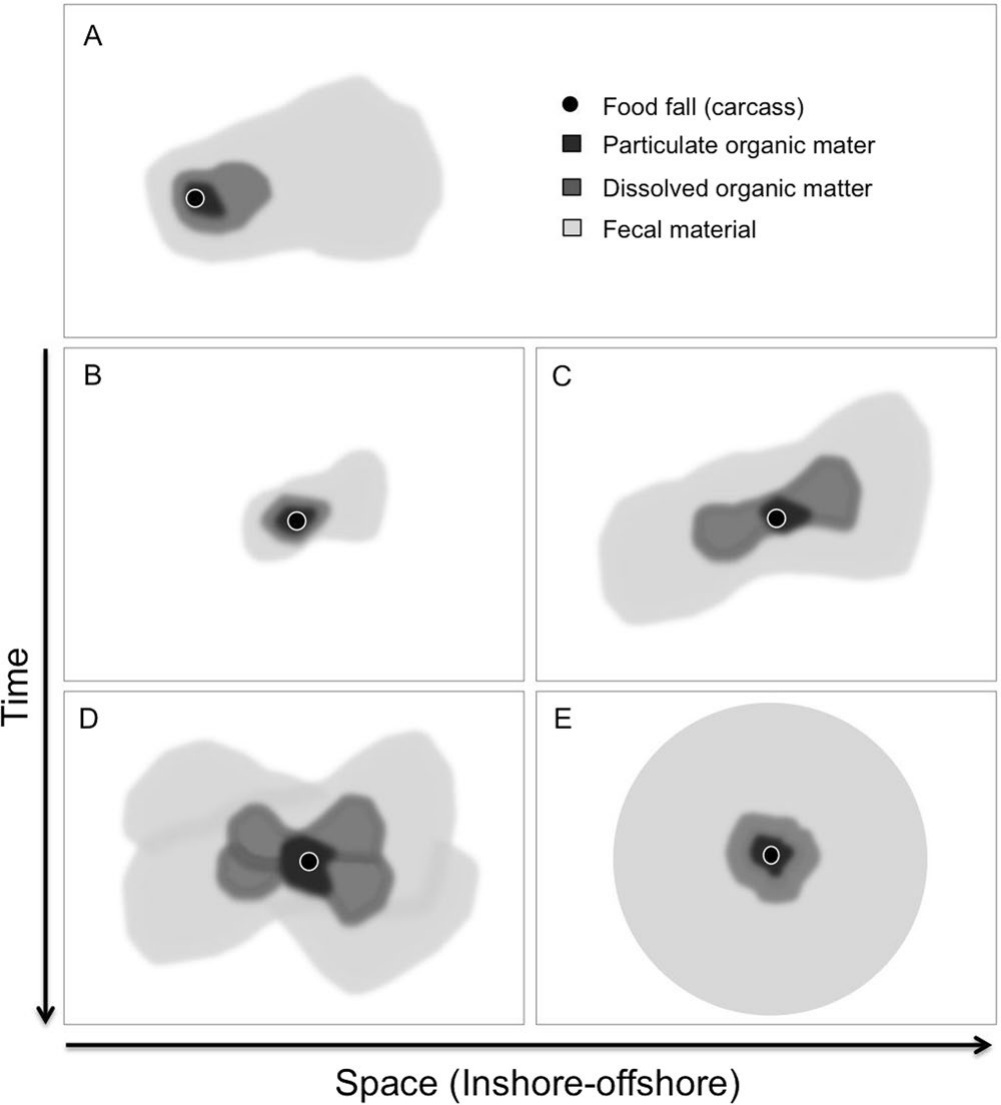
Conceptual model diagram of spatial footprint of organic matter dispersed from a punctual food-fall source (i.e., whale carcass). (**A**) Model proposed by^16^. Under the model assumption, the overall asymmetry of the spatial footprint is related to the asymmetry of currents along the bottom. Additionally, the area of influence of dissolved organic material is controlled by the lower limit of detection by benthic and demersal organisms; with fecal material being dispersed by highly mobile animals, and reaching greater distances from the center of the food fall. (**B**–**E**) Four hypothetical sequential stages of a proposed model that includes species-specific behavioral rhythms controlled by day-night and tidal cycles and affecting organic matter dispersal. In this slightly modified model, asymmetry occurs only at a first stage in the downstream current direction (**A**). Over time span of days to months, highly mobile scavengers and predators moving along the main bidirectional tidal currents^17^,^22^,^34^,^35^, split the asymmetry initially at the main bi-directional current flow (E–W in our present study; **C**). (**D**) Over longer times scales, from months to a year, depending on the initial mass of the carcass, the spatial footprint would become homogeneous in all directions due to large faunal displacements along and across depth contours (**B**).

In conclusion, our study brings some new insights about the temporal dynamics of community succession around whale fall habitats. Using combined high-frequency time-lapse photography and oceanographic data acquisition, we were able to show how species-specific behavioral rhythms influence community composition at shorter (diel, and weekly) time-scales near the carcass. Additionally, we obtained evidences supporting the occurrence of niche partitioning based on species interactions that may play a significant role in overall community succession and energy dispersal from whale carcasses into adjacent deep-sea open slope environments. Particularly, in light of our findings we propose a revision, or perhaps an addition to Stockton and Delaca’s conceptual model^16^ of energy dispersal from punctual sources of food falls in the deep seafloor (Fig. 7). Our new proposed model would encompass the effects of shorter time scale variability in faunal occurrence and activity near a large carcass due to day-night and tidally driven behavioral rhythms, which could further enhance the spatial footprint of energy dispersal in the form of particulate and dissolved organic matter, but more importantly, by fecal material released from species undergoing large rhythmic displacements along the slope and across wide depth ranges.

## Electronic supplementary material

Appendix 1-3
