# Supplementary material for: Faunal activity rhythms influencing early community succession of an implanted whale carcass offshore Sagami Bay, Japan
Source: Sci Rep. 2018 Jul 24;8:11163. doi: 10.1038/s41598-018-29431-5 (PMC6057991; doi:10.1038/s41598-018-29431-5)

Supplementary information to:

Faunal activity rhythms influencing early community succession of an implanted whale carcass offshore Sagami Bay, Japan

J. Aguzzi, E. Fanelli, T. Ciuffardi, A. Schirone, F.C. De Leo, C. Doya, M. Kawato, M. Miyazaki, Y. Furushima, C. Costa, and Y. Fujiwara

Appendix 1. Seventy-two-days' time-series of visual counts for the different megafaunal taxonomical units identified during the scavenging process of the sunken whale carcass (following the same taxonomical order as in Figure 2). Vertical dashed grey lines indicate time when the whale carcass changed its position.

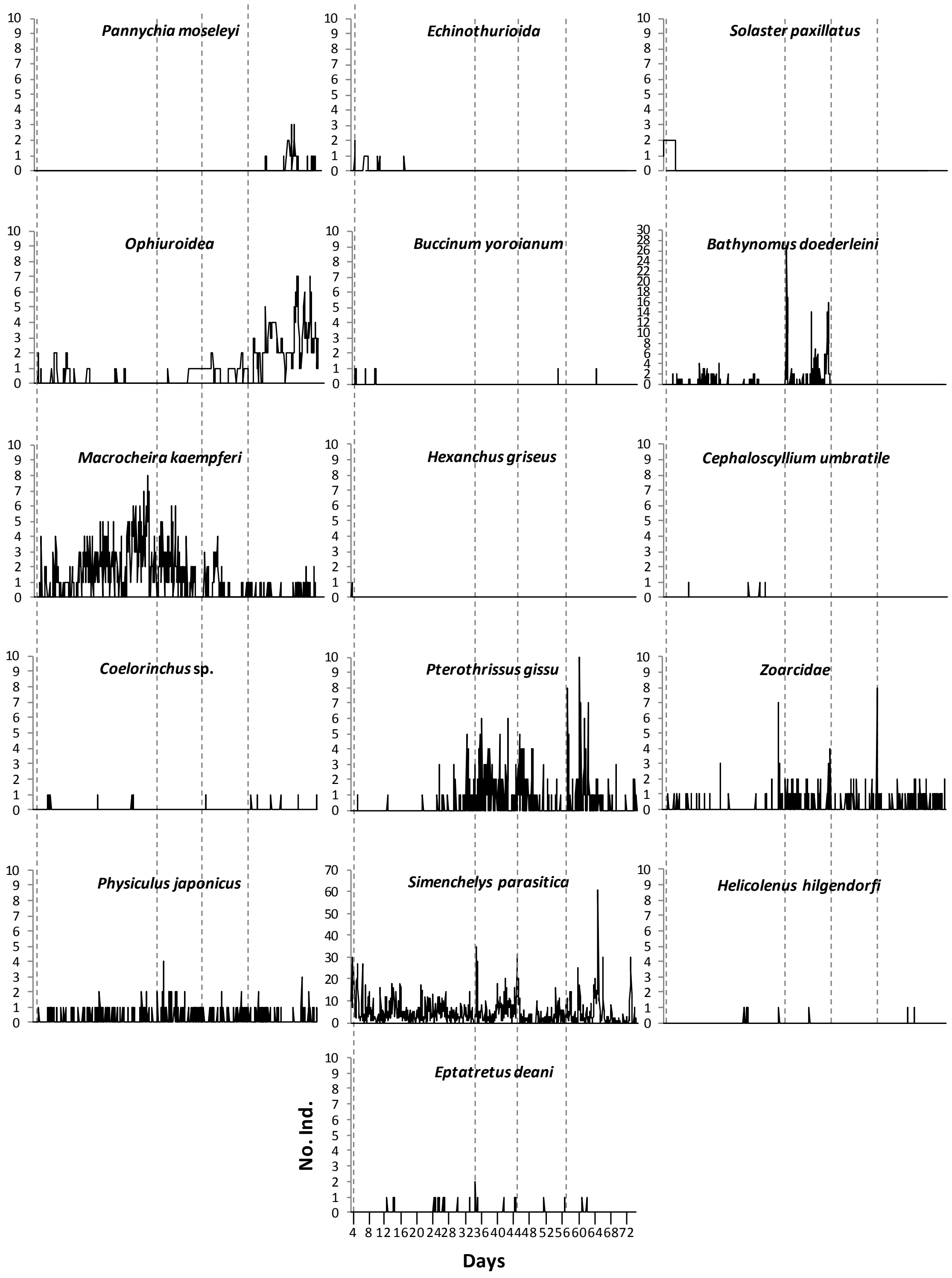

Appendix 2.

A. Time series of vertical current data from the ADCP, at 5, 32, 62, and finally 92 m above sea bottom, as computed by applying a 25 h moving average window. Dominant negative values indicate prominent downward flow in each layer, especially close to the bottom.

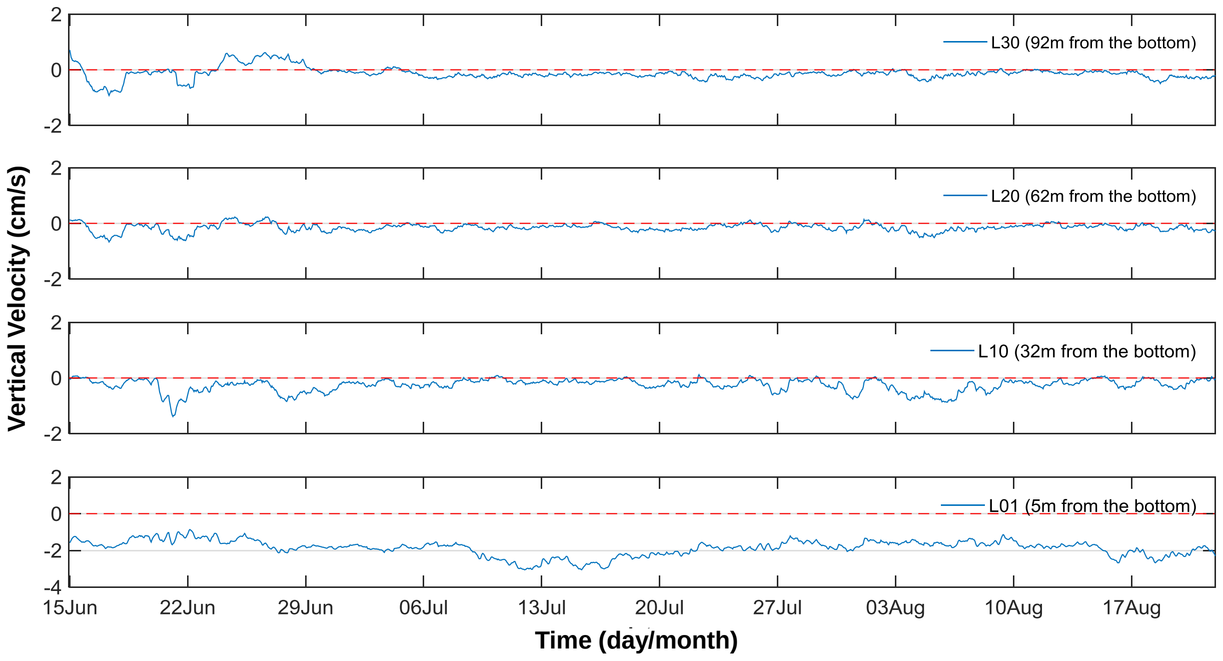

B. Power Spectra of water temperature, East-West, North-South and Vertical components of the velocity current data from the ADCP, at 5 m above sea bottom. Spectra of current data clearly show peak periods for 24, 12, and 6-hour, whereas peaks for temperature data are only evident at 12 and 6 h. The flow near the sea bottom is affected by tidal periodicity. This rhythm may affect the behavior of marine organism.

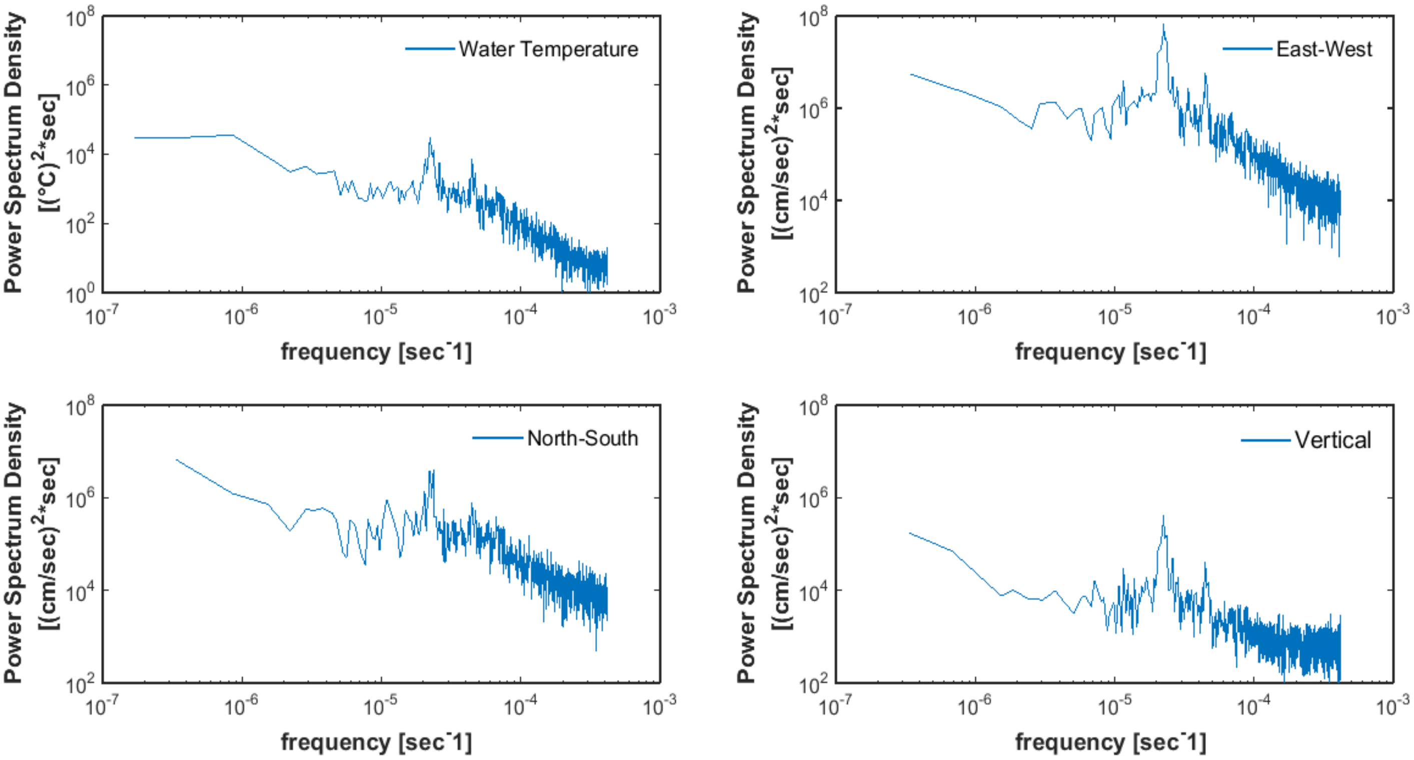

Appendix 3.

A. Cluster Analysis based on mean abundance (i.e., visual count) values by day of all species recorded at the whale carcass, and "week" as the samples. Black lines indicate significant sample clusters at p<0.05 according to SIMPROF test.

B. nMDS plots superimposed with results from the cluster analysis (at 60% of resemblance level) of two of the dominant taxa of each phase (weeks 1-6 vs. 7-11): *Macrocheira kaempferi* and ophiuroids. Bubbles represent the mean abundance of the species in each week.

a)

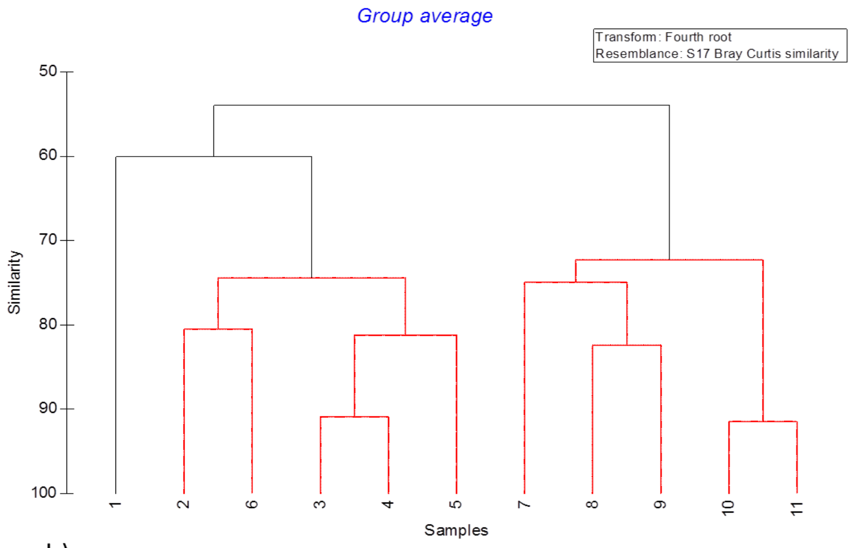

b)

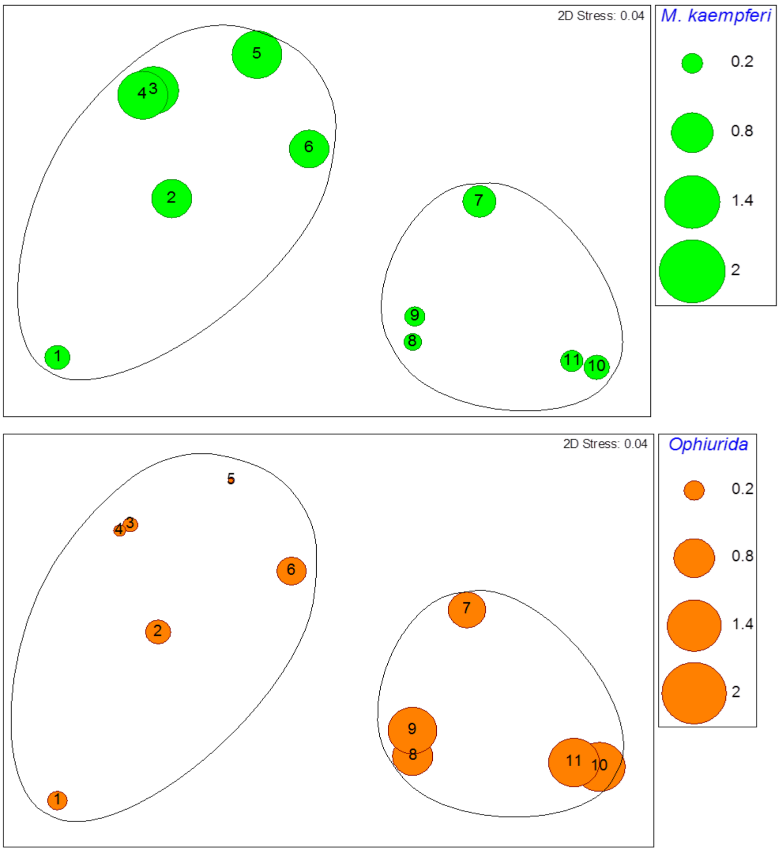

Supplement: Supplementary file 1 — Appendix 1-3 [file 41598_2018_29431_MOESM1_ESM.pdf]
